# Inflammatory M1-like macrophages polarized by NK-4 undergo enhanced phenotypic switching to an anti-inflammatory M2-like phenotype upon co-culture with apoptotic cells

**DOI:** 10.1186/s12950-020-00267-z

**Published:** 2021-01-07

**Authors:** Keizo Kohno, Satomi Koya-Miyata, Akira Harashima, Takahiko Tsukuda, Masataka Katakami, Toshio Ariyasu, Shimpei Ushio, Kanso Iwaki

**Affiliations:** 1 Pharma Medical Section, Healthcare Products Unit, Hayashibara Co., Ltd., Okayama, Japan; 2 Sales Development Section, Wellness Products Unit, Hayashibara Co., Ltd., Okayama, Japan; 3 R&D Division, Hayashibara Co., Ltd, Okayama, Japan

**Keywords:** Inflammation, Macrophage polarization, Phagocytosis, Phenotypic switching, Wound healing

## Abstract

**Background:**

NK-4 has been used to promote wound healing since the early-1950s; however, the mechanism of action of NK-4 is unknown. In this study, we examined whether NK-4 exerts a regulatory effect on macrophages, which play multiple roles during wound healing from the initial inflammatory phase until the tissue regeneration phase.

**Results:**

NK-4 treatment of THP-1 macrophages induced morphological features characteristic of classically-activated M1 macrophages, an inflammatory cytokine profile, and increased expression of the M1 macrophage-associated molecules CD38 and CD86. Interestingly, NK-4 augmented TNF-α production by THP-1 macrophages in combination with LPS, Pam3CSK4, or poly(I:C). Furthermore, NK-4 treatment enhanced THP-1 macrophage phagocytosis of latex beads. These results indicate that NK-4 drives macrophage polarization toward an inflammatory M1-like phenotype with increased phagocytic activity.

Efferocytosis is a crucial event for resolution of the inflammatory phase in wound healing. NK-4-treated THP-1 macrophages co-cultured with apoptotic Jurkat E6.1 (Apo-J) cells switched from an M1-like phenotype to an M2-like phenotype, as seen in the inverted ratio of TNF-α to IL-10 produced in response to LPS. We identified two separate mechanisms that are involved in this phenotypic switch. First, recognition of phosphatidylserine molecules on Apo-J cells by THP-1 macrophages downregulates TNF-α production. Second, phagocytosis of Apo-J cells by THP-1 macrophages and activation of PI3K/Akt signaling pathway upregulates IL-10 production.

**Conclusion:**

It is postulated that the phenotypic switch from a proinflammatory M1-like phenotype to an anti-inflammatory M2-like phenotype is dysregulated due to impaired efferocytosis of apoptotic neutrophils at the wound site. Our results demonstrate that NK-4 improves phagocytosis of apoptotic cells, suggesting its potential as a therapeutic strategy to resolve sustained inflammation in chronic wounds.

## Background

Macrophages play an essential role in the first line of defense against invading pathogens. Macrophages polarize toward two phenotypes, classically activated (M1) and alternatively activated (M2), depending on the microenvironment conditions [1]. In vitro activation with proinflammatory cytokines, such as interferon gamma (IFN-γ), tumor necrosis factor-α (TNF-α), and interleukin-12 (IL-12), and pathogen-associated molecular patterns (PAMPs), such as lipopolysaccharide (LPS), polarizes M1 macrophages. M1 macrophages release proinflammatory cytokines, such as IL-1, IL-6 and TNF-α, and are involved in inflammatory responses against intracellular pathogens, upregulation of Th1 responses, and tumoricidal activity [1, 2]. Thus, strategies to polarize macrophages towards an M1-like phenotype may be useful for protection against intracellular pathogens and for upregulating anti-tumor activity. However, due to their toxic nature, caution should be taken when applying proinflammatory cytokines and bacterial PAMPs in vivo. Therefore, it is essential to develop an immunomodulator with low or no side effects to promote polarization toward M1-like macrophages.

In contrast, macrophages polarize toward an M2 phenotype in response to IL-4, IL-10, or IL-13 in vitro. M2 macrophages release high levels of IL-10 and low levels of proinflammatory cytokines and are involved in parasite clearance, dampening inflammation, and wound healing [1, 2]. Furthermore, increased numbers of M2 macrophages are present in the airways of patients with allergic asthma [3]. During airway inflammation, IL-33 is produced by airway epithelial cells after antigen challenge, which modulates M2 macrophage polarization through suppression of tumorigenicity 2 (ST2) [4]. M2 macrophages are further divided into 3 subpopulations depending on specific stimulators [5, 6].

M2 macrophages are essential for the resolution of inflammation and wound healing. In the early phase of acute wound healing, inflammatory M1 macrophages and polymorphonuclear neutrophils clear pathogens and debris. After sanitization of the wound, neutrophils die by apoptosis. Inflammatory M1 macrophages then phagocytize apoptotic neutrophils, a process called efferocytosis, which signals for the resolution of inflammation and a phenotypic shift to an anti-inflammatory M2 phenotype with wound healing capacity [6]. However, in chronic wounds, such as pressure, venous, and diabetic ulcers, the shift from an M1 to M2 phenotype is dysregulated, and inflammatory M1 macrophages predominate at the chronic wound margin [6, 7]. The global annual increase in the number of patients suffering from pressure and venous ulcers indicates a need for therapeutic approaches to restore impaired efferocytosis in chronic wounds [7].

NK-4 (IUPAC name: 1-ethyl-4-[(1Z,3E,5E)-1-(1-ethylquinolin-1-ium-4-yl)-5-(1-ethylquinolin-4-ylidene)penta-1,3-dien-3-yl]quinolin-1-ium;iodide) is a divalent cationic pentamethine trinuclear cyanine dye that contains three quinolinium rings, N-ethyl side chains, and two iodine anions [8]. NK-4 is the common name of cryptocyanine O.A.1 (PubChem CID: 5489539). We recently demonstrated that NK-4 abrogated the IL-4-driven cytokine production profile in human monocytic THP-1 cells from proinflammatory to anti-inflammatory [8]. In the current study, we investigated whether NK-4 polarizes phorbol 12-myristate 13-acetate (PMA)-differentiated THP-1 cells with a macrophage-like phenotype (THP-1 macrophages) toward an M1-like phenotype. We found that NK-4 by itself or in synergy with TLR agonists polarized THP-1 macrophages to an M1-like phenotype with potent phagocytic activity. Interestingly, when NK-4-treated THP-1 macrophages were co-cultured with apoptotic cells, we observed phenotypic switch to an M2-like phenotype, as demonstrated by an inverted ratio of TNF-α to IL-10 following LPS stimulation. These results are consistent with the plasticity of macrophages and provide a mechanistic rationale for the potential use of NK-4 in wound healing.

## Results

### NK-4 polarizes THP-1 macrophages towards an M1-like phenotype with enhanced phagocytic activity

**Fig. 1:**
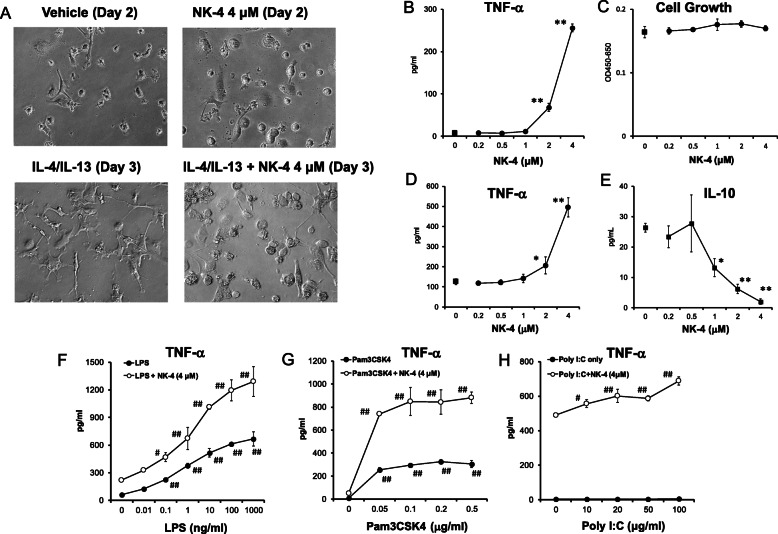
NK-4 polarizes THP-1 macrophages toward an M1-like phenotype based on morphology and cytokine production. THP-1 macrophages were cultured with various concentrations of NK-4 and/or with 20 ng/ml IL-4/IL-13 for 2–3 days. Morphological features were assessed by light microscopy (**a**). Levels of TNF-α at day 3 were measured in culture supernatants by ELISA (**b**). Cell numbers at day 3 were determined by cell counting kit-8 (**c**). 3 days post-NK-4 treatment, cells were stimulated with 1 μg/ml LPS for 2 days. Levels of TNF-α (d) and IL-10 (**e**) were measured in the culture supernatants by ELISA. THP-1 macrophages were stimulated with various concentrations of LPS (f), Pam3CSK4 (**g**), or poly(I:C) (**h**) in the presence or absence of 4 μM NK-4 for 1–3 days. Levels of TNF-α in the culture supernatants on day 1 (**f, g**) and day 3 (**h**) were determined by ELISA. Graphs show the mean ± S.D. of triplicate cultures and are representative of three independent experiments with similar results. *p < 0.05, **p < 0.01 compared with control cultures. #*p* < 0.5, ##*p* < 0.01 compared with cultures without stimulant for closed circles and cultures stimulated with NK-4 only for open circles

We first determined whether NK-4 polarizes THP-1 macrophages towards an M1-like phenotype. We examined the morphological features of THP-1 macrophages cultured with NK-4 or vehicle (control) in the presence or absence of IL-4 and IL-13 (IL-4/IL-13) by light microscopy. In contrast to the control cells, which displayed various morphologies, the majority of NK-4-treated THP-1 macrophages were round with increased size and granularity after 2 days of culture. These morphological features are consistent with those of human M1 macrophages polarized from blood monocytes with a combination of GM-CSF, IFN-γ, and LPS [9]. In contrast, THP-1 macrophages cultured with only IL-4/IL-13 for 3 days had a spindle-shaped appearance consistent with those of human M2 macrophages polarized from blood monocytes treated with a combination of M-CSF and IL-4 [9]. Interestingly, THP-1 macrophages cultured with both NK-4 and IL-4/IL-13 for 3 days predominantly exhibited M1-like morphological features (Fig. 1a). These results suggest that NK-4 polarizes THP-1 macrophages towards an M1-like phenotype and that the NK-4-induced signals are dominant to those induced by IL-4/IL-13.

We next assessed the functionality of NK-4-treated THP-1 macrophages by analyzing cytokine production. NK-4 dose-dependently increased THP-1 macrophage secretion of the proinflammatory cytokine TNF-α after 3 days of culture (Fig. 1b). At 4 μM NK-4, TNF-α secretion increased by 36-fold compared to control cells (Fig. 1b). NK-4 did not induce the secretion of anti-inflammatory cytokine IL-10 (data not shown). We did not detect endotoxin in 100 μM of NK-4 using a chromogenic assay, suggesting that the increase in TNF-α secretion by NK-4 was not due to endotoxin contamination (data not shown). NK-4 treatment did not affect THP-1 macrophage cell growth (Fig. 1c). We next analyzed cytokine secretion by NK-4-treated THP-1 macrophages following LPS stimulation. NK-4 dose-dependently increased TNF-α production and decreased IL-10 production (Fig. 1d, e).

As IFN-γ in combination with bacterial and viral PAMPs, such as LPS, Pam_3_Cys-Ser-(Lys)_4_ (Pam3CSK4), and poly(I:C) induces tumoricidal M1-like macrophages [10], we next examined the effects of combined treatment of NK-4 with these PAMPs on TNF-α production. Both LPS and Pam3CSK4 dose-dependently stimulated THP-1 macrophages to secrete TNF-α independent of NK-4 (Fig. 1f, g). Interestingly, NK-4 synergized with LPS and Pam3CSK4 to induce TNF-α production by THP-1 macrophages (Fig. 1f, g). Furthermore, NK-4 induced TNF-α production by THP-1 macrophages cultured with poly(I:C), although culture with poly(I:C) alone did not stimulate TNF-α production (Fig. 1h). IL-10 production was not detected in cultures treated with both NK-4 and LPS (data not shown).

**Fig. 2:**
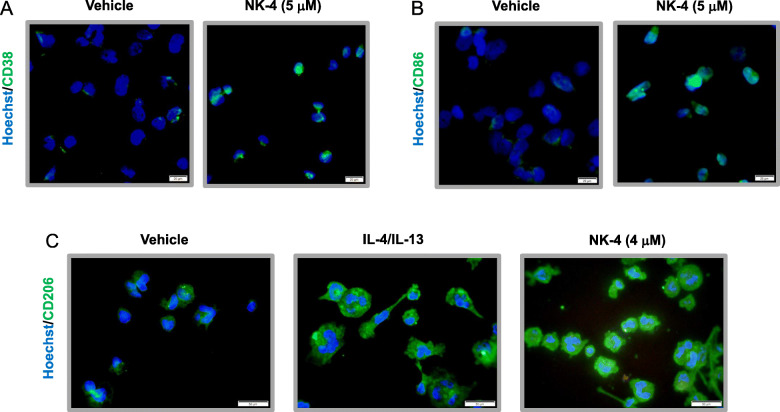
NK-4 upregulates CD38, CD86, and CD206 expression on THP-1 macrophages. THP-1 macrophages were cultured with 4–5 μM NK-4 for 3 days. Cells were then fixed and stained for CD38 (**a**), CD86 (**b**), or CD206 (**c**) using specific antibodies (green). Nuclei were detected with Hoechst 33258 (blue). Results are representative of three independent experiments with similar results

We next analyzed the cell-surface expression of M1 and M2 macrophage-associated surface antigens. NK-4 treatment increased the expression of the M1 macrophage-associated markers CD38 and CD86 [6, 7, 9, 11, 12] on THP-1 macrophages compared to control cells (Fig. 2a, b). We then examined the effect of NK-4 on the expression of CD206, a proposed M2 macrophage-associated marker [7, 9], in THP-1 macrophages. Consistent with previous reports, co-culture of THP-1 macrophages with IL-4/IL-13 enhanced the expression of CD206 compared to control cells (Fig. 2c). Surprisingly, NK-4-treated THP-1 macrophages also exhibited increased expression of CD206 compared to control cells (Fig. 2c).

**Fig. 3:**
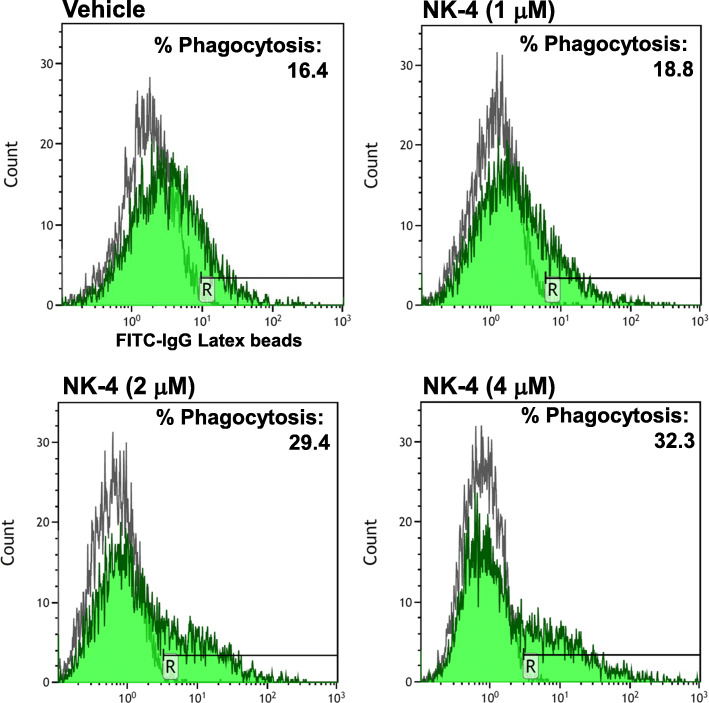
NK-4 enhances THP-1 macrophage phagocytosis. THP-1 macrophages were cultured with various concentrations of NK-4 for 3 days. Cells were recovered, and phagocytosis of rabbit IgG-FITC conjugated latex beads was analyzed by flow cytometry. Results are expressed as the percent phagocytosis and are representative of two independent experiments with similar results

We then examined the phagocytic activity of NK-4-treated THP-1 macrophages using rabbit IgG-FITC conjugated latex beads. As shown in Fig. 3, the percent of THP-1 macrophages that had phagocytosed beads increased as the concentrations of NK-4 increased. At the highest dose of 4 μM NK-4, the percent phagocytosis increased as much as 200% compared to control cells (Fig. 3).

Together, the observed changes in morphology, surface marker expression, and cytokine expression profiles suggest that NK-4 polarizes THP-1 macrophages toward an M1-like phenotype with enhanced phagocytic activity. However, the increased expression of CD206 in NK-4-treated THP-1 macrophages is surprising and requires further investigation.

### NK-4-treated inflammatory M1-like macrophages undergo a phenotypic switch to an anti-inflammatory M2-like phenotype upon co-culture with apoptotic cells

**Fig. 4:**
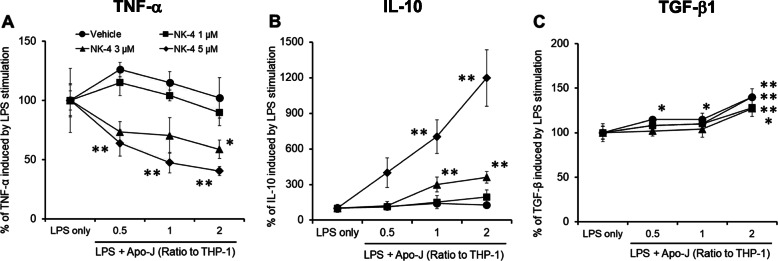
Phenotypic switch from an NK-4-induced M1-like to an M2-like phenotype upon co-culture with Apo-J cells. NK-4-treated THP-1 macrophages were co-cultured with Apo-J cells at various ratios for 1 h. Mixed cells were then stimulated with 1 μg/ml LPS for 2 days. Levels of TNF-α (**a**), IL-10 (**b**), and TGF-β1 (**c**) were measured in the culture supernatants by ELISA. Results are given as changes in cytokine levels relative to the mean values of the cells stimulated with LPS alone. Graphs show the mean ± S.D. of triplicate cultures and are representative of three independent experiments with similar results. *p < 0.05, **p < 0.01 compared with control cultures

In acute wound healing, successful efferocytosis of apoptotic neutrophils by macrophages transmits signals for the resolution of inflammation, resulting in a phenotypic switch from the inflammatory M1 phenotype to the anti-inflammatory M2 phenotype with wound healing capacity [6, 7]. We hypothesized that NK-4-treated M1-like THP-1 macrophages would undergo a phenotypic switch to an M2-like phenotype after co-culture with apoptotic cells. To test this hypothesis, we treated Jurkat E6.1 cells with H_2_O_2_ to induce apoptosis [13] and cultured NK-4-treated THP-1 macrophages alone or with apoptotic Jurkat E6.1 (Apo-J) cells at various ratios. After co-culture, we stimulated the cells with LPS and analyzed the levels of TNF-α, IL-10, and TGF-β1 in the supernatant. Apo-J cells dose-dependently decreased TNF-α production and increased IL-10 production by THP-1 macrophages treated with 3 and 5 μM NK-4 (Fig. 4a, b). Apo-J cells alone did not produce TNF-α or IL-10 following LPS stimulation (data not shown). There was no association between the levels of TGF-β1 and doses of NK-4.

We calculated the ratios of TNF-α to IL-10 from the data obtained in Fig. 4, which are summarized in Table 1. In the absence of Apo-J cells, the ratio of TNF-α to IL-10 increased in correlation with the doses of NK-4. At a concentration of 5 μM NK-4, the ratio of TNF-α to IL-10 increased as much as 100-fold compared to control cells. These results confirm the above findings that NK-4 by itself dose-dependently polarizes THP-1 macrophages toward an M1-like phenotype.

**Table 1:**
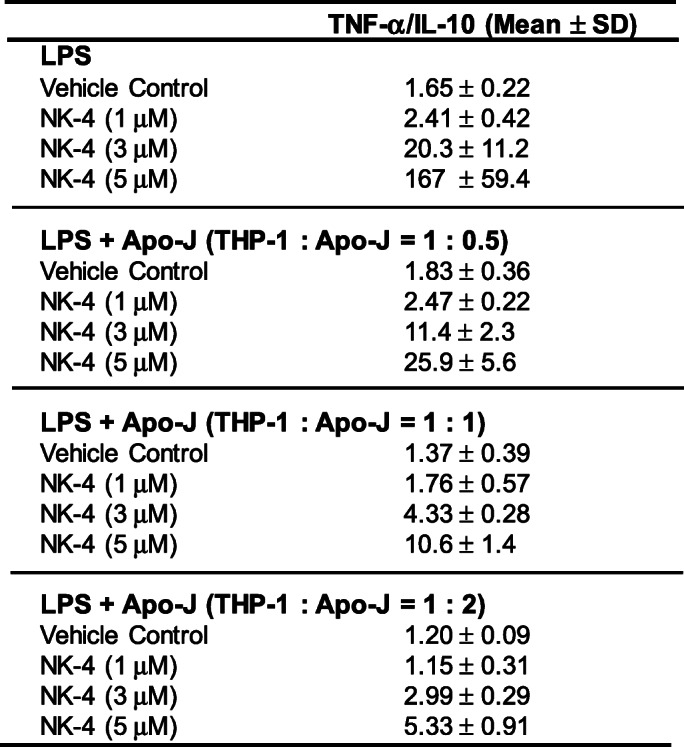
Ratios of TNF-α/IL-10 levels in all cultures examined in Fig. 4

Ratios of TNF-α/IL-10 levels in all cultures examined in Fig. 4 were calculated and expressed as the means ± S.D. of triplicate cultures.

However, the ratio of TNF-α to IL-10 decreased as the number of THP-1 macrophages relative to Apo-J cells decreased. At THP-1 to Apo-J ratios of 2:1, 1:1, and 1:2, treatment with 5 μM NK-4 reduced the ratio of TNF-α to IL-10 to 15.5, 6.3 and 3.2% of those with NK-4 treatment alone, respectively (Table 1).

### Mechanism of action for the phenotypic switch from NK-4-induced M1-like macrophages to an M2-like phenotype after co-culture with the apoptotic cells

We hypothesized that phosphatidylserine recognition on Apo-J cells is required for the phenotypic switch of NK-4-treated THP-1 macrophages, as the recognition of phosphatidylserine is the first key step in phagocytosis of apoptotic cells [14]. To test this hypothesis, Apo-J cells were pretreated with annexin V to mask the function of phosphatidylserine molecules before co-culture with NK-4-treated THP-1 macrophages. NK-4-treated THP-1 macrophages co-cultured with Apo-J cells produced 40% less TNF-α following LPS stimulation compared to NK-4-treated THP-1 macrophages alone (

**Fig. 5:**
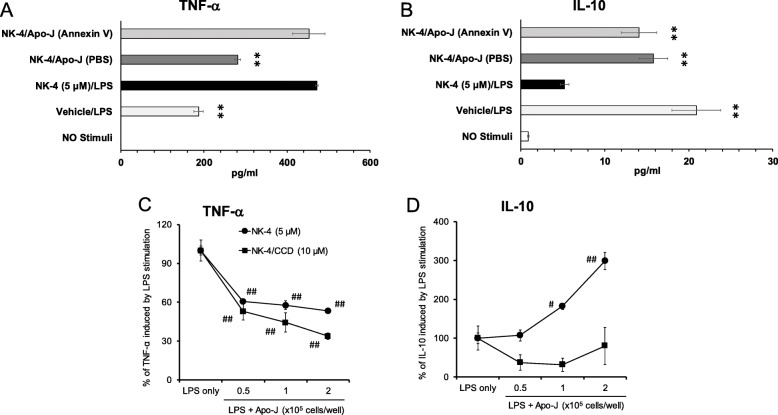
Reduction in TNF-α and increase in IL-10 are independently regulated during phagocytosis of Apo-J cells. Apo-J cells were pretreated with 10 μg/ml annexin V before co-culture with NK-4 (5 μM)-treated THP-1 macrophages at a 1:1 ratio (**a, b**). NK-4 (5 μM)-treated THP-1 macrophages were pretreated with cytochalasin D (CCD) before co-culture with Apo-J cells at a 1:1 ratio (**c, d**). TNF-α (**a, c**) and IL-10 (**b, d**) levels were measured by ELISA. Bar graphs show levels of cytokines (mean ± SEM, *n* = 3) (**a, b**). Line graphs show percent change from baseline levels of cytokines produced in response to LPS alone (mean ± SEM, n = 3) (**c, d**). Results are representative of two independent experiments with similar results. **p < 0.01 compared with cells stimulated with NK-4 and LPS in the absence of Apo-J. #*p* < 0.05, ##*p* < 0.01 compared with cells stimulated with LPS alone

Fig. 5a). However, pre-treatment of Apo-J cells with annexin V completely abrogated the reduction in TNF-α production (Fig. 5a). Interestingly, although co-culture of NK-4-treated THP-1 macrophages with Apo-J cells increased IL-10 production, annexin V did not affect IL-10 production (Fig. 5b).

Next, we examined whether phagocytosis of Apo-J cells upregulates IL-10 production by NK-4-treated THP-1 macrophages. We pretreated macrophages with cytochalasin D, which blocks > 90% of phagocytosis [15], before co-culture with Apo-J cells. Pretreatment with cytochalasin D did not affect TNF-α secretion, but completely abrogated the increase in IL-10 (Fig. 5c, d). These results suggest that the phagocytosis of Apo-J cells signal NK-4-treated THP-1 macrophages to upregulate IL-10 production.

**Fig. 6:**
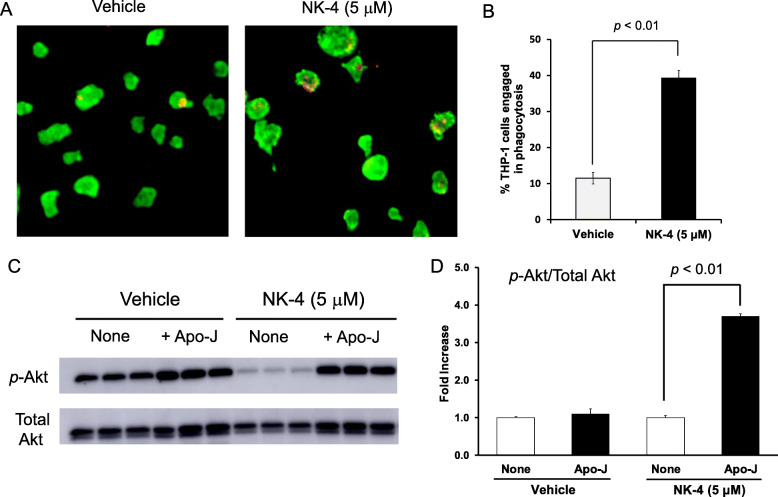
Phagocytosis of Apo-J cells by NK-4-treated THP-1 macrophages induces Akt activation. NK-4 (5 μM)-treated THP-1 macrophages were co-cultured for 1 h with Cell Tracker Red-stained Apo-J cells at a 1:1 ratio, followed by immunostaining for CD86 (**a**). The frequency of macrophages engaged in phagocytosis of Apo-J cells is shown (**b**). Phosphorylation of Akt (Ser473) in NK-4 (5 μM)-treated THP-1 macrophages incubated with or without Apo-J cells was determined by western blot. A representative blot is shown (**c**). The optical density ratio of phospho-Akt to total Akt is shown (**d**). Graphs show the mean ± SEM of triplicate cultures and are representative of two independent experiments with similar results

To determine whether NK-4 treatment promoted the phagocytosis of Apo-J cells by THP-1 macrophages, we co-cultured NK-4-treated THP-1 macrophages with Cell Tracker Red-stained Apo-J cells and analyzed phagocytosis by immunostaining. NK-4 increased the frequency of phagocytic THP-1 macrophages by 3.4-fold compared to vehicle control macrophages (Fig. 6a, b), demonstrating that NK-4 treatment promotes the phagocytosis of Apo-J cells by THP-1 macrophages.

As previous studies demonstrated that phagocytosis is associated with a rapid increase in phosphatidylinositol 3-kinase (PI3K)/Akt phosphorylation [16], we analyzed the phosphorylation of Akt in THP-1 macrophages that were co-cultured in the presence or absence of Apo-J cells by Western blotting. Co-culture of NK-4-treated THP-1 macrophages with Apo-J cells increased the phosphorylation of Akt, and the p-Akt to total Akt ratio was 3.7-fold higher compared to NK-4-treated THP-1 macrophages alone (Fig. 6c, d). In the vehicle-treated THP-1 macrophages, phosphorylation of Akt appeared to increase by co-culture with Apo-J cells (Fig. 6c). However, there was no significant increase in the ratio of p-Akt/total Akt (Fig. 6d).

**Fig. 7:**
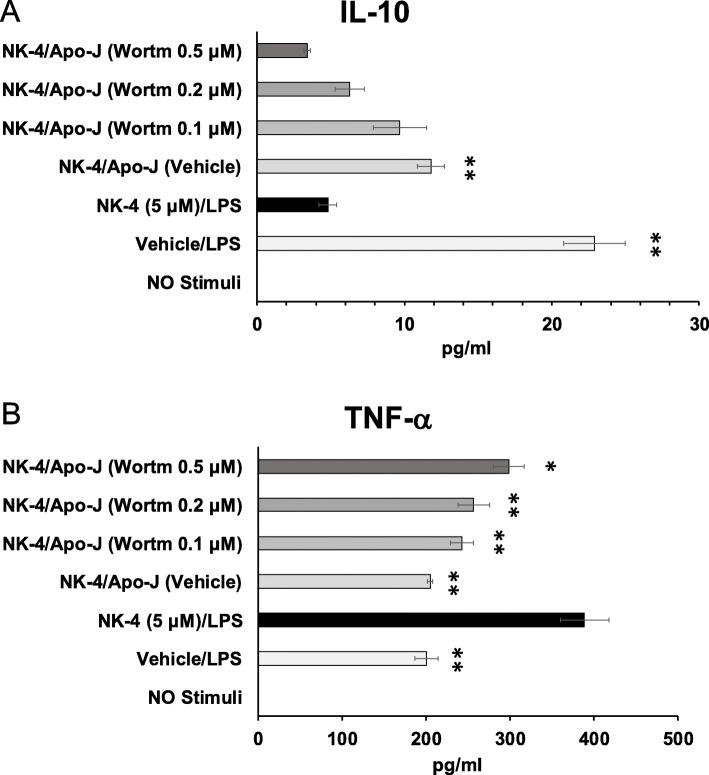
NK-4-treated THP-1 macrophages upregulate IL-10 production after Apo-J phagocytosis via the PI3K/Akt signaling pathway. NK-4 (5 μM)-treated THP-1 macrophages were pretreated with wortmannin before co-culture with Apo-J cells for 1 h at a 1:1 ratio. Mixed cells were then stimulated with 1 μg/ml LPS for 2 days. Levels of IL-10 (**a**) and TNF-α (**b**) were measured in the culture supernatants by ELISA. Graphs show the mean ± SEM of triplicate cultures and are representative of two independent experiments with similar results. *p < 0.05, **p < 0.01 compared with cells stimulated with NK-4 and LPS in the absence of Apo-J

We hypothesized that the PI3K/Akt signaling pathway contributed to the upregulation of LPS-stimulated IL-10 production by NK-4-treated THP-1 macrophages following phagocytosis of Apo-J cells. To test this hypothesis, we pretreated NK-4-treated THP-1 macrophages with wortmannin, a PI3K inhibitor, before co-culture with Apo-J cells. Wortmannin dose-dependently down-regulated IL-10 secretion by NK-4-treated THP-1 macrophages, with a concentration of 0.5 μM wortmannin completely inhibiting the increase in IL-10 (Fig. 7a). Pretreatment with wortmannin resulted in partial recovery of LPS-stimulated TNF-α production (Fig. 7b).

## Discussion

NK-4 has been used to promote wound healing since the early-1950s; however, the mechanism of action remains unknown. Macrophages play a key role at all stages of wound healing from the initial inflammatory process after injury until the tissue regeneration process [6, 7]. At the wound site, macrophages are activated and polarized toward an inflammatory M1 phenotype in response to proinflammatory cytokines and PAMPs [1, 2]. M1 macrophages promote the phagocytosis of pathogens and the removal of damaged cells, including neutrophils.

In this study, we showed that NK-4 polarizes THP-1 macrophages toward the inflammatory M1-like phenotype in terms of morphological features, increased ratio of TNF-α to IL-10 produced after LPS stimulation, and increased expression of M1-macrophage-associated molecules CD38 and CD86. Furthermore, we found that NK-4 treatment enhanced THP-1 macrophage phagocytosis of latex beads.

Interestingly, we found that NK-4 enhanced TNF-α production by THP-1 macrophages in combination with LPS, Pam3CSK4, and poly(I:C). A previous study found that proinflammatory cytokines, such as TNF-α and IL-8, prime the neutrophil oxidative burst [17]. Furthermore, LPS stimulation of NK-4-treated THP-1 macrophages enhanced CD86 expression and phagocytosis of latex beads compared to either agent alone (data not shown). These results suggest that NK-4 promotes the polarization of wound macrophages toward an M1-like phenotype synergistically with pathogen-derived molecules and potentiates the first line of host defense against invaded pathogens during the inflammatory process of wound healing.

The mechanism by which NK-4 polarizes THP-1 macrophages toward a proinflammatory M1-like phenotype is unknown. A recent study showed that palmitic acid synergizes with LPS to induce MCP-1 production by RAW264.7 cells via the mitogen-activated protein kinase (MAPK)-mediated TLR4 signaling pathway [18]. In a preliminary study, we examined the effects of extracellular signal-regulated kinase (ERK), p38, and Jun N-terminal kinase (JNK) MAPK inhibitors on TNF-α production by THP-1 macrophages in response to NK-4. We found that U0126, an ERK1/2 inhibitor, significantly and dose-dependently inhibited TNF-α production by NK-4-treated THP-1 macrophages (data not shown). These results suggest that the ERK MAPK signaling pathway is involved in NK-4-induced TNF-α production. However, future studies are necessary to address whether the ERK MAPK signaling pathway is required for the morphological changes, upregulation of surface marker expression, and phagocytosis induced by NK-4.

Efferocytosis is essential to promote the resolution of inflammation [6, 7, 14]. The recognition and engulfment of apoptotic cells transmits immunosuppressive signals to macrophages. Voll et al. first demonstrated that the co-culture of LPS-activated peripheral blood mononuclear cells and monocytes with apoptotic peripheral blood lymphocytes inhibited TNF-α production and increased release of IL-10 [19]. In our study, NK-4-treated THP-1 macrophages produced significantly less TNF-α and more IL-10 in response to LPS in the presence of Apo-J cells. We did not observe a significant difference in TGF-β1 production between vehicle control and NK-4-treated THP-1 macrophages. These results indicate that inflammatory M1-like THP-1 macrophages polarized by NK-4 underwent phenotypic switching to an anti-inflammatory M2-like phenotype after co-culture with apoptotic cells.

We think that NK-4 is not directly involved in switching to the M2-like phenotype. Since THP-1 macrophages pretreated with NK-4 for 3 days were washed twice before co-culture with Apo-J cells, NK-4 could be removed from the co-culture system. We showed that NK-4 treatment enhanced THP-1 macrophage phagocytosis of rabbit IgG-FITC conjugated latex beads. Because of this increased phagocytic activity, we consider that NK-4-pretreated THP-1 macrophages could efficiently phagocytose Apo-J cells, resulting in the phenotypic switch from M1-like to M2-like phenotype.

In this study, we demonstrated that at least two separate mechanisms are involved in the phenotypic shift from the inflammatory M1-like to the anti-inflammatory M2-like phenotype. First, recognition of phosphatidylserine molecules on Apo-J cells by THP-1 macrophages down-regulates TNF-α production. Second, the phagocytosis of Apo-J cells upregulates IL-10 production. Although we have not identified phosphatidylserine receptors on THP-1 macrophages, TAM receptor family proteins (Tyro 3, Axl, and MerTK) may be possible candidates, as their cognate ligands, growth arrest-specific protein 6 (Gas6) and protein S (ProS1), have direct anti-inflammatory activity that suppresses NF-κB and inflammatory cytokines independently of efferocytosis [20, 21]. Furthermore, it was previously shown that the binding of apoptotic cells to the surface of phagocytes is sufficient for the down-regulation of inflammatory cytokines [22]. These results support our findings that the down-regulation of LPS-stimulated TNF-α production by co-culture with Apo-J cells occurred even when cytochalasin D inhibited phagocytosis.

THP-1 macrophages upregulated IL-10 production following LPS stimulation after phagocytosis of Apo-J cells. We demonstrated that activation of the PI3K/Akt signaling pathway is involved in this process, as described previously [16]. The ratio of p-Akt/total Akt significantly increased in NK-4-treated THP-1 macrophages after co-culture with Apo-J cells. Furthermore, pre-treatment of NK-4-treated THP-1 macrophages with wortmannin completely abrogated the upregulation of IL-10 production. Vehicle-treated THP-1 macrophages phagocytosed Apo-J cells to some extent, but culture with Apo-J cells did not significantly increase the p-Akt/total Akt ratio. These results suggest that phagocytosis that leads to distinct activation of the PI3K/Akt signaling pathway is necessary for the upregulation of IL-10 production.

Activation of the PI3K/Akt signaling pathway causes inhibitory phosphorylation of the downstream signaling molecule, glycogen synthase kinase 3 (GSK3) [23]. It was previously shown that inhibitory phosphorylation of GSK3 enhanced IL-10 production by human monocytes stimulated with TLR2, TLR4, TLR5, or TLR9 agonists while suppressing the secretion of proinflammatory cytokines [24]. GSK3 phosphorylation upregulates IL-10 production and down-regulates the proinflammatory cytokines by augmenting cAMP response element binding protein (CREB) binding to the nuclear coactivator, CREB-binding protein (CBP), which in turn suppresses the binding of NF-κB p65 to CBP [24]. We hypothesize that inhibition of the PI3K/Akt/GSK3 pathway may explain the partial recovery of TNF-α production by NK-4-treated THP-1 macrophages after wortmannin treatment.

The numbers of patients with chronic wounds, such as diabetic, venous and pressure ulcers, are rising annually and globally, resulting in a substantial economic burden in developed countries [7]. Furthermore, wounds in aged or diabetic patients are refractory to treatments and can become chronic [25]. The most postulated pathogenic mechanism for chronic wounds is that the switch from a proinflammatory M1 macrophage to an anti-inflammatory M2 phenotype is dysregulated due to impaired efferocytosis of apoptotic neutrophils at the wound site [6, 7, 14]. The persistent presence of proinflammatory M1 macrophages and inefficient apoptotic cell clearance, which eventually progresses to secondary necrosis and the release of proinflammatory intracellular contents, prolongs the inflammatory phase [6, 26, 27]. In addition, dysfunctional macrophage efferocytosis impairs the resolution of inflammation in the wounds of diabetic mice [28]. Thus, strategies to enhance macrophage efferocytosis are necessary for the resolution of chronic wounds.

In this study, we showed that NK-4-treated THP-1 macrophages exhibited enhanced phagocytosis of apoptotic cells as well as IgG-coated latex beads. Upon phagocytosis of apoptotic cells, the LPS-stimulated cytokine production profile was changed from a proinflammatory to an anti-inflammatory response, as seen in the inverted ratio of TNF-α to IL-10. These results suggest that NK-4 could promote the resolution of sustained inflammation in chronic wounds by augmenting macrophage efferocytosis. Our results further support the idea that an individual macrophage is engaged sequentially in both the induction and the resolution of inflammation [29].

There are numerous reports showing increased efferocytic capacity of M2 macrophages [30–32]. In contrast, several reports describe high levels of phagocytic activity of M1 macrophages [1, 6, 7]. These discrepancies may be partially explained by differences in efferocytic activity of M1 macrophages between acute and chronic inflammatory conditions. During the early phase of acute wound healing, macrophages accumulated in wounds are activated by inflammatory cytokines and PAMPs and are polarized toward a M1 phenotype. These M1 macrophages phagocytose apoptotic neutrophils and remove pathogens and cellular debris in the wounds [1, 6, 7]. In this respect, M1 macrophages are highly efferocytic. In chronic wounds, an increased and prolonged inflammatory phase is commonly observed [6]. Furthermore, macrophages are composed predominantly of the M1 phenotypes that are unable to phagocytose apoptotic neutrophils at the chronic wound margin [6, 33]. Thus, the efferocytic capacity of inflammatory M1 macrophages in chronic wounds appears to be impaired. These results further suggest that M1 macrophages do not always retain potent efferocytic activity in vivo.

On the other hand, the reason why M2 macrophages have increased efferocytic capacity may be that phagocytic macrophages are still needed to remove remaining apoptotic cells resulting from the inflammatory environment after switching to the M2 phenotype. Recently, it has been shown that M2 macrophages that contribute to the final stages of wound healing are synergistically activated by both Th2 cytokines and apoptotic cell engulfment [34]. Further studies are necessary to explain the discrepancies.

Surprisingly, NK-4 increased the expression of the M2 macrophage-associated marker CD206 on THP-1 macrophages. CD206 is a mannose receptor (MR) with C-type lectin domains. The MR is predominantly expressed by most tissue macrophages and recognizes intracellular pathogens such as *Mycobacterium tuberculosis* and *Leishmania* species [35]. As CD206 expression is increased on macrophages by treatment with IL-4/IL-13, it is recognized as an M2 macrophage-associated marker [36]. However, CD206 is also expressed by tissue-resident macrophages in both mice and humans [37] and can be maintained in the absence of IL-4 receptors [38]. Furthermore, tissue-resident macrophages with phagocytic function can express CD206 [39]. We recently demonstrated that NK-4 suppresses the STAT6 signaling pathway in human dermal fibroblasts stimulated with IL-4 and TNF-α [8]. Together, these results suggest that NK-4 increased CD206 expression in THP-1 macrophages independently of IL-4/STAT6 signals.

Billions of apoptotic cells are eliminated by tissue-resident phagocytic macrophages every day from a healthy body without causing inappropriate inflammation or an immune response [39]. Our findings that NK-4 increased CD206 expression and phagocytic activity in THP-1 macrophages suggest that NK-4 may contribute to maintaining tissue homeostasis, although future studies are necessary to test this hypothesis.

## Conclusions

We demonstrated that NK-4 drove macrophage polarization toward an inflammatory M1-like phenotype with enhanced phagocytic activity, suggesting that NK-4 potentiates the first line of host defense against invaded pathogens during the inflammatory phase of wound healing. Furthermore, we showed that NK-4-induced M1-like macrophages undergo elevated phenotypic switching toward an anti-inflammatory M2-like phenotype upon co-culture with apoptotic cells. Our results suggest that NK-4 may provide a promising strategy for the resolution of chronic wounds.

## Methods

### Reagents

NK-4 was synthesized at Functional Dyes Unit, Hayashibara Co., Ltd. (Okayama Japan). A stock solution of 10 mM NK-4 was prepared with DMSO and stored frozen at − 80 °C. Endotoxin content in 100 μM NK-4 was below detection limits (0.00625 endotoxin units/ml), as determined according to a protocol listed in the Japanese Pharmacopoeia using an Endospecy ES-50 M set (Seikagaku Co., Tokyo, Japan). Final concentrations of DMSO at 0.05% or less did not affect the results of the experiments. PMA and LPS (*E. coli* O55:B5) were purchased from Sigma-Aldrich Japan (Tokyo, Japan). Pam3CSK4 hydrochloride was purchased from InvivoGen (San Diego, CA). Poly(I:C) was purchased from Calbiochem (La Jolla, CA). Recombinant human IL-4 and IL-13 were purchased from R&D Systems (Minneapolis, MN). Recombinant annexin V was purchased from BD Biosciences (Franklin Lakes, NJ). Wortmannin was purchased from Wako Pure Chemical (Osaka, Japan). Human TNF-α, and monoclonal antibodies (mAb) for the human TNF-α ELISA were prepared and purified in our laboratories.

### Culture and differentiation of THP-1 cells

The human monocytic cell line, THP-1 (ATCC, Manassas, VA), was maintained in complete medium comprised of RPMI 1640 (Sigma-Aldrich) supplemented with 10% FCS (GE Healthcare Life Sciences, South Logan, UT) and 1% Penicillin-Streptomycin (Wako Pure Chemical) in a 5% CO_2_ humidified atmosphere at 37 °C.

For differentiation to a macrophage phenotype, THP-1 cells (1.1 × 10^5^ /well) were incubated with 100 nM PMA in 48-well tissue culture plates (Corning, Kennebunk, ME) at 37 °C for 2 days. Following differentiation, PMA-containing media was replaced with complete medium and cells were rested for 24 h.

### Stimulation of THP-1 macrophages

After the resting period, THP-1 macrophages were washed once with RPMI1640 medium supplemented with 1% FCS and 1% Penicillin-Streptomycin (conditioned medium) and incubated with 4 μM NK-4, unless otherwise stated, and/or with 20 ng/ml human IL-4 and IL-13 at 37 °C for 2–3 days. After treatment with NK-4 for 3 days, cells were washed twice with conditioned medium and stimulated with 1 μg/ml LPS for 2 days. In some experiments, THP-1 macrophages were stimulated with various concentrations of LPS, Pam3CSK4, or poly(I:C) in the presence or absence of 4 μM NK-4 at 37 °C for 1–3 days.

Morphological features of THP-1 macrophages were examined by an inverted microscope (ECLIPSE TS100, Nikon, Tokyo, Japan).

Cell growth was assessed by cell counting kit-8 (Wako Pure Chemical, Osaka, Japan). Briefly, 15 μl WST-8 reagent, a redox indicator, was added to each well for the last 2 to 3 h of the incubation period. The optical density of the culture supernatants was measured at 450 nm.

### Immunostaining

THP-1 cells were seeded at 7.5 × 10^4^ cells per well in the 8-well chamber slide (LAB-TEK, Rochester, NY) and were differentiated as described above. THP-1 macrophages were cultured with 4 or 5 μM NK-4 or with 20 ng/mL human IL-4/IL-13 in conditioned medium for 3 days at 37 °C. Cells were fixed with 4% paraformaldehyde in PBS for 15 min at room temperature, washed and permeabilized with 0.1% (v/v) Triton X-100 for 30 min. After overnight incubation in PBS containing 3% BSA at 4 °C, cells were treated with human FcR blocking reagent (Miltenyi Biotech, Auburn, CA) and were stained with the following antibodies: FITC-labelled mouse anti-human CD38 mAb (303504, BioLegend CNS, Inc., San Diego, CA), FITC-labelled mouse anti-human CD86 mAb (555657, BD PharMingen), Alexa Fluor 488-labelled mouse anti-human CD206 mAb (FAB25342G, R&D Systems). Nuclei were detected with Hoechst 33258. Stained cells were detected with an inverted fluorescence microscope (BX53F-B, OLYMPUS, Tokyo, Japan).

### Phagocytosis of rabbit IgG-FITC conjugated latex beads

3 days after incubation with varying concentrations (0, 1, 2, or 4 μM) of NK-4, cells were recovered by pipetting with ice-cold phosphate-buffered saline (PBS) containing 5 mM EDTA and were washed once with conditioned medium. The cells were then assessed for their phagocytic activity using a Phagocytosis Assay kit (Cayman Chemical, Ann Arbor, MI). Briefly, latex beads with rabbit IgG-FITC conjugates (1:100) were incubated with vehicle- or NK-4-treated cells for 2 h at 37 °C, followed by 2 min of incubation with trypan blue to quench non-phagocytosed bead fluorescence. Cells were washed twice with PBS containing 0.1% bovine serum albumin (BSA) and analyzed by flow cytometry (Gallios, Beckman Coulter Japan, Tokyo). The percent phagocytosis was expressed as the proportion of THP-1 macrophages that phagocytosed fluorescent beads.

### Analysis of the phenotypic switch from M1-like to M2-like THP-1 macrophages after co-culture with apoptotic cells

THP-1 macrophages were treated with various concentrations (0, 1, 3, or 5 μM) of NK-4 for 3 days in 48-well tissue culture plates as described above. After the treatment, cells were washed two times with the conditioned medium, and fresh medium was added to each well.

Jurkat E6.1 (ATCC) cells were suspended at 1 × 10^6^ cells/ml in complete medium and treated with 50 μM H_2_O_2_ for 6 h at 37 °C to specifically induce apoptosis as described previously [13]. Cells were washed three times with the conditioned medium, suspended in the same medium, and counted with trypan blue. Apoptotic Jurkat E6.1 (Apo-J) cells were then added to the culture of NK-4-pretreated THP-1 macrophages at various ratios, and the mixed cells were incubated for 1 h at 37 °C. Since cell growth of THP-1 macrophages was comparable between control and NK-4 cultures after 3 days of treatment (Fig. 1c), the number of THP-1 macrophages (1.1 × 10^5^ /well) added to each well of 48-well plates before PMA treatment was used when calculating the ratios. After the incubation period, the mixed cells were stimulated with 1 μg/ml LPS for 2 days at 37 °C. Levels of TNF-α at day 1 and levels of IL-10 and TGF-β1 on day 2 were measured by ELISA in the culture supernatants.

In some experiments, Apo-J cells were pretreated with 10 μg/ml annexin V for 30 min at 37 °C before co-culture with NK-4 (5 μM)-treated THP-1 macrophages. In other experiments, NK-4 (5 μM)-treated THP-1 macrophages were pretreated with 10 μM cytochalasin D or wortmannin (1–5 μM) for 30 min before co-culture with Apo-J cells.

### Cytokine assays

Cytokines (human TNF-α, IL-10 and TGF-β1) in culture supernatants were measured by two-site sandwich ELISA. Levels of human TNF-α were determined by a sandwich ELISA system that was developed in our laboratory. Briefly, this ELISA system utilizes mouse anti-TNF-α mAb (MAb-TNFα-5) and HRPO-conjugated mouse anti-TNF-α mAb (MAb-TNFα-1) for capture and detection of human TNF-α, respectively. The detection limit is 3 pg/ml. Levels of human IL-10 were determined with a human IL-10 ELISA set (Diaclone SAS, Cedex, France). Human TGF-β1 levels were determined with human TGF-β1 DuoSet ELISA Development Systems (DY240–05, R&D Systems, Minneapolis, MN).

### Phagocytosis of Apo-J cells

Jurkat E6.1 cells (1 × 10^6^ cells/ml) were stained with 7 μM Cell Tracker Red (excitation 577/emission 602) (Thermo Fisher Scientific, Waltham, MA) in serum-free RPMI1640 medium for 30 min at 37 °C. Cells were washed twice with complete medium, and apoptosis was induced in the Cell Tracker Red-treated Jurkat E6.1 cells by treatment with 50 μM H_2_O_2_ as described above. The Cell Tracker Red-treated Apo-J cells were incubated with 5 μM NK-4-treated THP-1 macrophages at a 1:1 ratio in conditioned medium for 1 h at 37 °C in the 4-well chamber slide (LAB-TEK). After the incubation period, the mixed cells were washed sequentially by complete medium and PBS to remove non-phagocytosed Apo-J cells. Cells were then fixed with 4% paraformaldehyde in PBS for 15 min at room temperature, washed and permeabilized with 0.1% (v/v) Triton X-100 for 30 min. After overnight incubation in PBS containing 3% BSA at 4 °C, THP-1 macrophages were treated with human FcR blocking reagent and were stained with FITC-labelled mouse anti-human CD86 mAb. The phagocytosis percentage was calculated in 5 random fields per each well. The number of macrophages that engulfed Apo-J cells in each field was counted and then divided by the total number of THP-1 macrophages in the same field. Results were expressed as mean ± SD of triplicate wells (15 fields in total).

### Western immunoblotting analysis of Akt

NK-4 (5 μM)-treated THP-1 macrophages were incubated with or without Apo-J cells at a ratio of 1:1 for 45 min at 37 °C. Whole-cell extracts were prepared with RIPA buffer (Wako Pure Chemical) containing phosphatase inhibitor (Nacalai Tesque Inc., Kyoto, Japan) and protease inhibitor (Roche Diagnostics, Mannheim, Germany) and subjected to western immunoblotting. Membranes were probed with a 1:1000 dilution of anti-phospho-Akt (Ser473) rabbit pAb (9171; Cell Signaling Technology, Danvers, MA). Specific bands were detected using an ECL™ Plus Western Blotting System (Immobilon Western Chemiluminescent HRP substrate; GE Healthcare, UK). After treatment with a reprobing solution (Restore Western Blot Stripping Buffer; Pierce Biotechnology, Rockford, IL) for 15 min at room temperature, the membrane was used for secondary detection with a 1:1000 dilution of anti-Akt rabbit mAb (C67E7; Cell Signaling Technology). Band density was measured using ImageQuant TL software (GE Healthcare).

### Statistical analysis

Statistical analyses were performed using Statcel 4 software (OMS Publishing Inc., Saitama, Japan). Phagocytic data were evaluated using the non-parametric Mann-Whitney U test. Other data analyses were conducted by one-way ANOVA followed by Dunnett’s test for multiple comparisons or by the Student t-test for comparison between two variables. *P* values < 0.05 were considered statistically significant.

## Data Availability

All relevant data are within the paper.

## Abbreviations

TNF-α: Tumor necrosis factor-α
PMA: Phorbol 12-myristate 13-acetate
Pam3CSK4: Pam_3_Cys-Ser-(Lys)_4_
LPS: Lipopolysaccharide
PAMPs: Pathogen-associated molecular patterns
Apo-J: Apoptotic Jurkat E6.1
PI3K: Phosphatidylinositol 3-kinase
MAPK: Mitogen-activated protein kinase
ERK: Extracellular signal-regulated kinase
GSK3: Glycogen synthase kinase 3
